# Western medical acupuncture is an alternative medicine or a conventional classic medical manipulation

**DOI:** 10.22088/cjim.10.2.239

**Published:** 2019

**Authors:** Ali Jabbari, Shabnam Tabasi, Jila Masrour-Roudsari

**Affiliations:** 1 Ischemic Disorders Research Center, Golestan University of Medical Sciences, Gorgan, Iran; 2 Department of Anesthesiology and Intensive Care Medicine, Golestan University of Medical Sciences, Gorgan, Iran; 3 Department of Hematology and oncology, Golestan University of Medical Sciences, shahid Sayad Shirazi Hospital, Gorgan, Iran; 4 Student Research Committee, Babol University of Medical Sciences, Babol, Iran

**Keywords:** Western medical acupuncture, alternative medicine, Traditional Chinese medicine

**Dear Editor,**

Traditional Chinese medicine is a practical medicine built on experience. Many years ago, the Chinese made a significant discovery. They found that needle insertion to the body can influence various functions of it, and they explained it by the theory that was acceptable at that time (1).

The Chinese had been employing a highly complex therapeutic procedure involving herbal medicine and the body needling. Insertion of needles into the body was called acupuncture. It was part of the traditional medicine in China for a long time. When acupuncture first arrived in the West; the principles which the Chinese had used to explain its actions were an encounter with current scientific knowledge of the humans' anatomies and physiology. This led to the denial of acupuncture by the medical profession. It was not commonly used and thus had little effect (1, 2).

As modern medicine’s impact increased, it led to the development of “integrative medicine”. Modern medicine has accepted the view that “nature that cannot be certified cannot be conquered,” but acupuncture based on traditional Chinese medicine propound itself as “integrative medicine”.The conceptual advances since the scientific revolution, particularly the relatively recent discoveries of the neurotransmitters and neuroplasticity, have lead to a new concept of the mechanisms of needling and justify the use of a new term, Western medical acupuncture (WMA). Western medical acupuncture is a therapeutic modality involving the insertion of fine needles. It is an adaptation of Chinese acupuncture using current knowledge of anatomy, physiology and pathology, and the principles of evidence based medicine. WMA is used to distinguish it from acupuncture used as part of Chinese traditional medicine (2).

 WMA involves the insertion of fine needles through the skin which is left in position for about 45 minutes and sometimes manual, electrical or thermal stimulation is applied to the needles. In western medical acupuncture; needling is seen as a form of neuromuscular stimulation that owes little to Chinese traditional meridians or points. In other words, Chinese traditional meridians and points were modified on the base of modern anatomical and physiological concept by a scientific person in WMA.WMA does not use the Chinese traditional concepts such as Yin-Yang and qi (1, 3).

In traditional Chinese philosophy, there are two principles, one negative and dark that is called feminine (yin) and one positive and bright called masculine (yang) whose interaction influences the destinies of creatures and human health. Based on Chinese philosophy; qi is the circulating life force whose existence and properties are dependent on its circulation (1-3).

The ideology which formed the basis of Chinese acupuncture has been discarded by medical practitioners in the Western world. Western medical acupuncture has come out from Chinese acupuncture; its practitioners no longer accept concepts such as Yin or Yang and circulation of qi and illustrate acupuncture as part of conventional modern medicine rather than an alternative medical system. “Same treatment for different diseases,” is one of the most important and characteristic concepts in traditional Chinese medicine but it is not accepted in WMA. Western medical acupuncturist believes that needle stimulation of the nervous system and its known modes of action include local antidromic axon reflexes, segmental and extra segmental neuromodulation, and other central nervous system effects (1, 2).

Unfortunately, the understanding of WMA is not uniform, and a number of variations are practiced, including minimally needling a restricted area, identify acupuncture treatment areas, subcutaneous needling over tender muscle trigger points or attempts to match the therapy completely conform with neurophysiological concepts. However, the current era of interest in the scientific approach to acupuncture to an influential, medically trained acupuncturist. Western medical acupuncture is principally used by healthcare practitioners, most commonly in primary care. It is mainly used to musculoskeletal pain relief especially the neck, shoulder and back. It is also effective for postoperative pain, migraine headache, anxiety and depression, menopausal problems, nausea and vomiting associated with chemotherapy. Its activity in these different problems and complications suggests that acupuncture does not have a single mode of action and there is a range of effects on various modalities which increases the complexity of both understanding and researching acupuncture (1-4).

Some western medical acupuncturists believe that acupuncture is as effective as or more effective than standard conventional care (2-5) but; when WMA is compared to sham needling, it often shows only a small positive trend. Western medical acupuncture practitioners explain this contradiction that may be expected since such studies are comparing two forms of sensory stimulation (2, 4).

It seems that western medical acupuncture practice acts like a form of treatment based on neurophysiological principles and for this reason, they consider their practice as a conventional medical method and they claimed WMA is a treatment that can relieve symptoms of some physical and psychological conditions and encourage the body to heal and repair itself, besides, some investigations show that acupuncture is superior to sham needling for specific problems (2-5) but in fact; they need more physiological and pathophysiological shreds of evidence for propounding itself as a routine conventional medical practice.

**Competing interests:**

One of the authors is participating in western medical acupuncture (WMA) training program, but there is no any kind of financial interest.
